# Postoperative thyroglobulin as a yard-stick for radioiodine therapy: decision tree analysis in a European multicenter series of 1317 patients with differentiated thyroid cancer

**DOI:** 10.1007/s00259-023-06239-8

**Published:** 2023-05-01

**Authors:** Luca Giovanella, Lisa Milan, Wolfgang Roll, Manuel Weber, Simone Schenke, Michael Kreissl, Alexis Vrachimis, Kim Pabst, Tuncel Murat, Petra Petranovic Ovcaricek, Burkhard Riemann, Luca Ceriani, Alfredo Campenni, Rainer Görges

**Affiliations:** 1 Clinic for Nuclear Medicine and Molecular Imaging, Imaging Institute of Southern Switzerland, Ente Ospedaliero Cantonale, Ospedale San Giovanni, Via A. Gallino 6, 6500 Bellinzona, CH, Switzerland; 2 Clinic for Nuclear Medicine, University Hospital Zürich, Zurich, Switzerland; 3 Clinic for Nuclear Medicine, University Hospital Münster, Münster, Germany; 4 Clinic for Nuclear Medicine, University Hospital Essen, Essen, Germany; 5 Clinic for Nuclear Medicine, University Hospital Magdeburg, Magdeburg, Germany; 6 Department of Nuclear Medicine, German Oncology Center, Limassol, Cyprus; 7 Department of Nuclear Medicine, Hacettepe University, Ankara, Turkey; 8 Department of Oncology and Nuclear Medicine, University Hospital Center “Sestre milosrdnice”, Zagreb, Croatia; 9 Nuclear Medicine Unit, Department of Biomedical and Dental Sciences and Morpho-Functional Imaging, University of Messina, Messina, Italy

**Keywords:** Differentiated thyroid cancer, DTC, Post-treatment whole-body scintigraphy, PT-WBS, Decision tree model, Thyroglobulin, Tg

## Abstract

**Purpose:**

An accurate postoperative assessment is pivotal to inform postoperative ^131^I treatment in patients with differentiated thyroid cancer (DTC). We developed a predictive model for post-treatment whole-body scintigraphy (PT-WBS) results (as a proxy for persistent disease) by adopting a decision tree model.

**Methods:**

Age, sex, histology, T stage, N stage, risk classes, remnant estimation, TSH, and Tg were identified as potential predictors and were put into regression algorithm (conditional inference tree, ctree) to develop a risk stratification model for predicting the presence of metastases in PT-WBS.

**Results:**

The lymph node (N) stage identified a partition of the population into two subgroups (N-positive vs N-negative). Among N-positive patients, a Tg value  > 23.3 ng/mL conferred a 83% probability to have metastatic disease compared to those with lower Tg values. Additionally, N-negative patients were further substratified in three subgroups with different risk rates according to their Tg values. The model remained stable and reproducible in the iterative process of cross validation.

**Conclusions:**

We developed a simple and robust decision tree model able to provide reliable informations on the probability of persistent/metastatic DTC after surgery. These information may guide post-surgery ^131^I administration and select patients requiring curative rather than adjuvant ^131^I therapy schedules.

## Introduction

A trend towards de-escalating the role of iodine-131 (^131^I) in the treatment of differentiated thyroid cancer (DTC) emerged after the release of the 2015 edition of the American Thyroid Association (ATA) Management Guidelines for adult patients with thyroid nodules and differentiated thyroid cancer [1]. Basically, a three-tiered postoperative risk assessment system, primarily based on pathology reports, was proposed to inform decisions on postoperative ^131^I administration (i.e., ATA risk classes). Such a system, however, cannot detect the presence of postoperative biochemical, structural, or functional persistent disease that require curative ^131^I administration. Moreover, different options (i.e., wait-and-see, remnant ablation, adjuvant ^131^I therapy) are available in patients with no evidence of persistent disease. ATA risk classes are relevant in assigning patients to different strategies but additional factors such as local resources and expertise as well as patient’ preferences should be incorporated and discussed, preferably in a multidisciplinary context (i.e., tumor board), in order to do justice to the modern concept of an individualized, targeted therapy [2]. The postoperative application of ^131^I, which encompasses the integration of diagnostics (i.e., post-treatment whole-body scintigraphy, PT-WBS) and therapeutics, served a long time as the gold standard for detecting persistent disease, assess its ^131^I avidity, and predict response to ^131^I, thus enabling personalized management of DTC patients. However, following current standards, the omission of ^131^I therapy in selected cases prevents the treating physician from obtaining such information; hence, alternative markers are warranted. Thyroglobulin (Tg) is a glycoprotein produced by the thyroid follicular cells roughly related to the amount of thyroid tissue present [3]. In DTC patients who undergo total thyroid ablation (i.e., thyroidectomy and ^131^I ablation), Tg is a powerful tumor marker and it is monitored to detect persistent or recurrent disease, evaluate disease progression, and provide prognostic informations, respectively [4]. Preablation Tg measurement can accurately determine the likelihood of achieving remission or having persistent or recurrent disease after the initial ^131^I therapy [5], and was also proposed to assess the postoperative status and guide ^131^I therapy selection with sparse results [6-8]. As a matter of fact, the predictive value of postoperative Tg is influenced by many factors. These include the time elapsed since surgery, the amount of thyroid remnant [9], the selected Tg cutoff level, and the TSH level at the time of Tg measurement [10-13]. Furthermore, the presence of Tg autoantibodies (TgAb), often “imported” by co-existing thyroid autoimmune diseases, significantly affects the measurable Tg values in up to 25% of DTC patients in the early postoperative period [3, 4, 14]. Accordingly, Tg reference intervals mathematically normalized to TSH levels and estimated amounts of thyroid remnants have been claimed to improve the reliability of postoperative Tg measurement [15, 16]. Moreover, the pre-test individual risk of loco-regional and distant metastases further complicates the issue. As a result, optimal Tg cutoff levels to distinguish normal residual thyroid tissue from persistent thyroid cancer requiring curative ^131^I administration are not yet available [1, 17, 18]. In the available literature, patients treated with ^131^I were predominantly grouped together as one cohort, irrespective of whether ^131^I was carried out in the context of remnant ablation, adjuvant therapy, or as therapy for known metastases. Considering new recommendations, however, early detection of patients with persistent disease after surgery is pivotal to optimize ^131^I treatment (i.e., patients’ preparation and administered ^31^I activities) to maximize the treatment effectiveness. First, metastatic DTC cells have lower density and poorer functionality of natrium iodine symporter (NIS) and the TSH elevation over time (i.e., area under the curve of TSH stimulation) is relevant to increase ^131^I uptake and retention [19]. Accordingly, thyroid hormone withdrawal (THW) is preferable to recombinant human TSH (rhTSH) in these cases (i.e., with the exception of patients who are either unable to elevate endogenous TSH during THW, or in whom THW is contraindicated for medical reason). Second, ablative and adjuvant treatments are performed with ^131^I activities ranging from 1.1 to 3.7 GBq while treatment of known disease is performed with higher activities ranging from 3.7 to 5.5 GBq for small-volume loco-regional disease and 5.6–7.4 GBq (150–200 mCi) or even more ^131^I for treatment of advanced locoregional disease and/or small-volume distant metastatic disease. Identification of iodine-avid diffuse metastatic disease may lead to escalation of prescribed therapeutic 131I activity with or without dosimetry calculations [20]. All in all, an accurate postoperative assessment is pivotal to inform our treatments and avoid suboptimal ^131^I administration (i.e., adjuvant instead of curative strategy). The present study was prompted to develop a predictive model for PT-WBS results (as a proxy for persistent disease) by adopting a decision tree model integrating postoperative TSH and Tg levels, thyroid remnant estimate, patients’ demographic and clinical data, and ATA risk classes, respectively, in a large series of 1317 TgAb-negative DTC patients.

## Material and methods

### Patients

From institutional databases of participating centers, all patients 18 years and older with histologically proved DTC who underwent (near-)/total thyroidectomy and THW-assisted ^131^I therapy were included. Records without information on (*i*) TgAb levels; (*ii*) preablation TSH, Tg, and 24-h radioiodine uptake (RAIU) values within 1 week before ^131^I therapy; and (*iii*) post-treatment whole-body scintigraphy (PT-WBS) results were excluded from the present study.

### Radioiodine treatment

Patients selected for ^131^I administration underwent THW and received a ^131^I activity determined at the discretion of the attending physician according to nuclear medicine guidelines and practice recommendations (median 2.4 GBq, range 1.1–4.5 GBq). Radioprotection issues were managed in strict adherence to national regulations [18, 21].

### RAIU testing and post-treatment WBS

All RAIU testing and PT-WBS examinations were performed strictly following the EANM procedure guidelines in all participating centers. Single photon emission computed tomography/computed tomography (SPECT/CT) was performed in addition to PT-WBS at the judgment of attending physicians [22]. The PT-WBS was classified as negative (i.e., absent uptake within the thyroid bed and absent non-physiological uptakes in other regions); remnant only (i.e., uptake within the thyroid bed without non-physiological uptakes in other regions); or positive (pathological uptake outside the thyroid bed, with or without uptake within the thyroid bed).

### Laboratory

Serum TSH levels were measured by 2nd or 3rd generation immunoassays on conventional automated analytical platforms. Assays employed to quantify serum Tg and TgAb are reported in Table 1.

**Table 1:** Thyroglobulin and thyroglobulin antibodies assays employed in different centers

Center	Tg assay	FS (ug/L)	TgAb assay	FS (UI/mL)	Tg-recovery
1	Kryptor® BRAHMS	0.15	Kryptor® BRAHMS	33	Yes
2*	Medizym® Tg Rem	0.09	Immulite® 2000 XPi	20	Yes
2*	SELco® Tg	0.3	Immulite® 2000 XPi	20	Yes
3	Elecsys® Tg	0.1	Elecsys® TgAb	40	Yes
4	Tg-plus® RIA BRAHMS	0.20	Varia	-	Yes
5	IRMA Cis Bio®	0.70	Elecsys® TgAb	40	No

*MedizymR Tg Rem for Tg values < 0.3 in SELcoR Tg

Legends, *Tg*, thyroglobulin; *TgAb*, thyroglobulin antibody; *RIA*, radioimmunoassay; *IRMA*, immunoradiometric assay; *FS*, functional sensitivity

### Decision tree model

Age, sex, histology, T stage, N stage, ATA risk, RAIU, TSH, and Tg were identified as potential predictors and were put into regression algorithm (conditional inference tree, ctree) to develop a risk stratification model for predicting the presence of metastasis in PT-WBS. Conditional inference tree analysis provides a decision tree by performing recursive population splitting into subgroups according to the specified clinical endpoint. At each partition, the algorithm searches for the best predictor and corresponding cut-off value that split the cohort into two subsequent nodes such that the outcome is significantly different between the two nodes, respectively. The process is iterated for each node until the algorithm cannot find any predictor that leads to significantly different subclasses, thus creating an algorithm for predicting future outcomes within more homogeneous subgroups. The final sets of subpopulations are called terminal nodes. The ctree algorithm allows to include both continuous and categorical variables in the analysis. Additionally, it ranks the  relevance of different variables instead of merely focusing on the outcome prediction with less attention to variables’ contribution. To minimize the overfitting, the dataset was divided into training and validation dataset through 200-fold cross validation after a preliminary choice of the most relevant variables. First, we evaluated the most frequently selected parameters by varying the dataset. To this end, the selection of patients to be included in the dataset was iterated 1000 times, maintaining the original proportion of variables and endpoint. The clinical parameters selected in at least the 95% of the iterations were used in the subsequent analysis. In a second step, these parameters were used to build the final model that was then validated by 200-fold cross-validation process applying a splitting ratio 70:30. The correct proportion of variables and events was maintained also in this case. For each iteration, the performance of the model was verified calculating accuracy, positive predictive value (PPV), and negative predictive value (NPV). Since for each iteration, the cutoff value of each node could slightly differ due to different patients; the median and interquartile range of threshold have been evaluated. Finally, the performance of the model was tested for each center to assess the impact of different Tg and TgAb assays.

### Statistical analysis

To analyze differences between different groups, *χ*^2^ test and Kruskal-Wallis or Mann-Whitney *U* tests were used for categorical and continuous variables, respectively. Differences were considered statistically significant when *P* ≤ 0.05. Statistical analyses were carried out with R and the integrated development environment R Studio v 1.2.1335 (RStudio, Inc., Boston, MA, USA). For the conditional inference tree analysis, the ctree function of the party R package was used.

## Results

Demographic, clinical, histopathological, and biochemical data included in our statistical model are summarized in Table 2 for the overall series and single series of different participating centers, respectively. Relevant between-center differences emerged for almost all parameters considered in our analysis (Table 2).

**Table 2:** Demographic, clinical, and pathological characteristics of DTC patient (overall population and populations of different participating centers)

Parameter	Overall (*n* = 1314)	Center 1 (*n* = 205)	Center2 (*n* = 208)	Center 3 (*n* = 83)	Center 4 (*n* = 371)	Center 5 (*n* = 447)	*P-value*
Age (years)	49 (40–59)	53 (46–65)	51.5 (39–64)	45 (38–55)	49 (41–57)	47 (38–58)	< 0.0001
> 55 years	430 (32.7%)	84 (41%)	87 (42%)	21 (25%)	108 (29%)	134 (30%)	0.0005**
Gender (M/F)	316/998	55/150	61/147	20/63	55/316	124/323	
	(24%/76%)	(27%/73%)	(29%/71%)	(24%/76%)	(15%/85%)	(28%/72%)	0.0001**
Histology							
PTC	827 (63%)	142 (69%)	94 (45%)	49 (59%)	186 (50%)	356 (79.5%)	
PTC-FV	233 (18%)	28 (14%)	60 (29%)	22 (27%)	122 (33%)	1 (0.5%)	
FTC	237 (18%)	35 (17%)	38 (18%)	11 (13%)	63 (17%)	90 (20%)	
HCTC	17 (1%)	-	16 (8%)	1 (1%)	-	-	< 0.0001**
TSH (µUI/mL)	64.5 (44–93)	70 (47–100)	66 (43–92)	41 (22–76)	70 (47–93)	63 (45–94)	< 0.0001*
TSH > 30 µUI/mL	1185 (90%)	185 (90%)	180 (86%)	55 (66%)	338 (91%)	427 (96%)	< 0.0001**
Tg (ng/mL)	3 (0.7–10)	3.4 (0.9–14.1)	2.9 (0.7–7.3)	5.8 (1.1–17)	2.8 (0.7–11.1)	2.7 (0.7–8.5)	0.034*
RAIU (%)	4.4 (2.2–7.8)	2 (1–3.3)	3.7 (2.1–7.1)	2.2 (1.3–4.3)	6.6 (3–12)	5.1 (3.3–7.5)	< 0.0001*
ATA risk							
Low	709 (54%)	80 (39%)	125 (60%)	49 (59%)	242 (65%)	213 (48%)	
Intermediate	346 (26%)	57 (28%)	33 (16%)	17 (20.5%)	105 (28%)	134 (30%)	
High	259 (21%)	68 (33%)	50 (24%)	17 (20.5%)	24 (7%)	100 (22%)	< 0.0001**
T stage							
1	693 (53%)	73 (36%)	105 (50%)	46 (55%)	236 (64%)	233 (52%)	
2	291 (22%)	57 (27%)	75 (36%)	22 (27%)	56 (15%)	81 (18%)	
3	313 (24%)	73 (36%)	24 (12%)	14 (17%)	69 (21%)	123 (28%)	
4	17 (1%)	2 (1%)	4 ( 2%)	1 (1%)	0 (0%)	10 (2%)	< 0.0001**
N stage							
0	1099 (84%)	139 (68%)	179 (86%)	69 (83%)	370 (99.7%)	342 (77%)	
1	215 (16%)	66 (32%)	29 (14%)	14 (17%)	1 (0.3%)	105 (23%)	
2	0 (0%)	0 (0%)	0 (0%)	0 (0%)	0 (0%)	0 (0%)	< 0.0001**
Positive PT-WBS	200 (15.2%)	42 (20.5%)	14 (6.7%)	5 (6%)	49 (13.2%)	90 (20.1%)	< 0.0001**

**Legend**: Continuous variables are expressed as median (interquartile range, *IQR*); proportions are expressed as percentage (%); *PTC*, papillary thyroid carcinoma; *PTC-FV*, papillary thyroid carcinoma-follicular variant; *FTC*, follicular thyroid carcinoma; *HCTC*, Hürtle cell thyroid carcinoma; *TSH*, thyroid stimulating hormone; *Tg*, thyroglobulin; *ATA*, American Thyroid Association; *RAIU*, 24-h radioiodine uptake; *T*, tumor; *N*, lymph node; *PT-WBS*, post-treatment whole-body scintigraphy

^*^Kruskal-Wallis test

^**^Chi square test

### Decision tree model

**Fig. 1:**
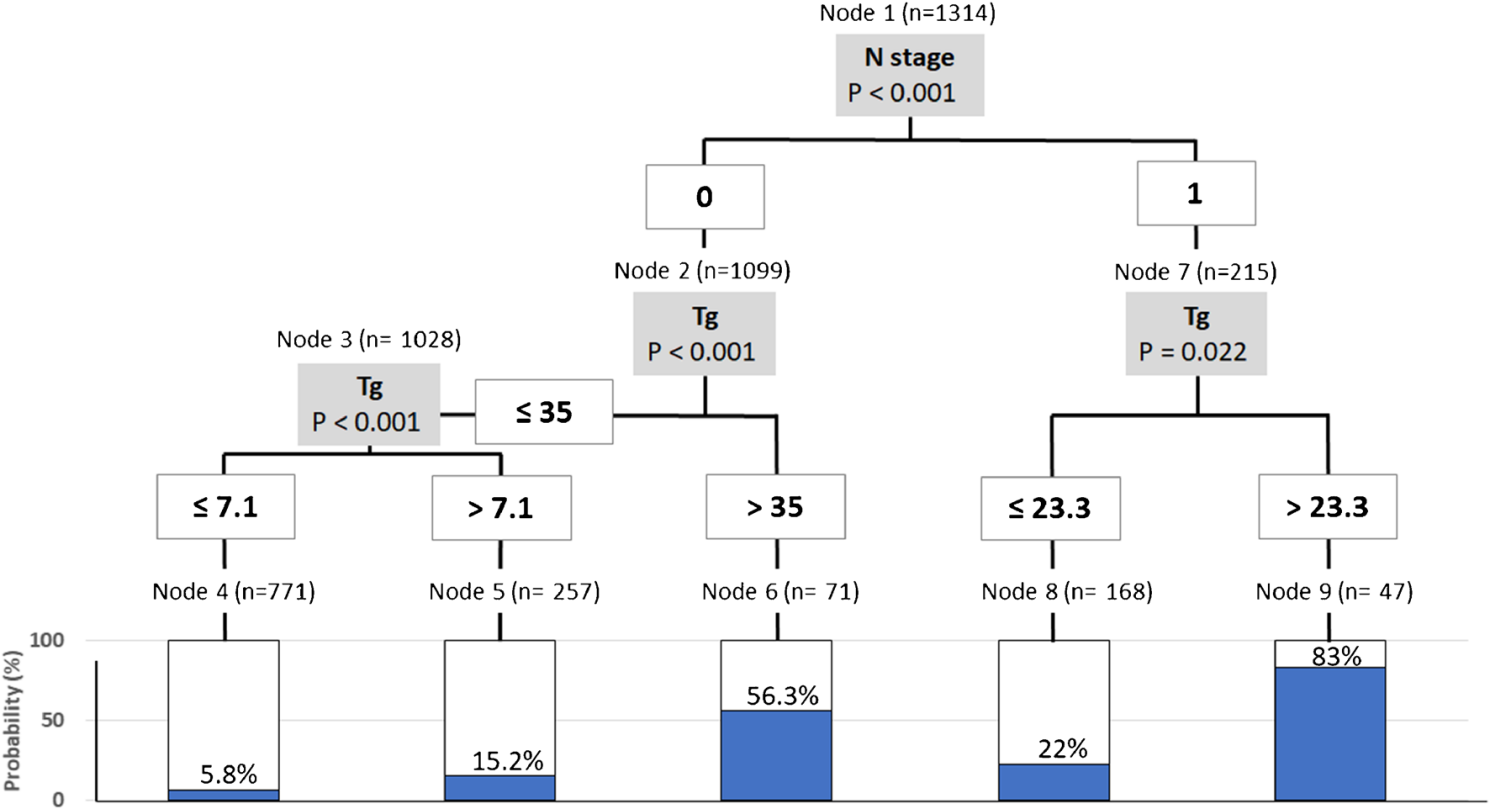
A graphical representation of classification tree: according to the decision tree model lymph node stage (N stage) subdivides the population into two subgroups. In case of lymph node involvement, patients with a value of Tg higher than 23.3 ng/mL carry a probability of 83% to have persistent/metastatic disease compared to those with lower Tg values. Patients without lymph node involvement are stratified in three-risk subgroups according to their Tg values: Tg values exceeding 35 ng/mL predict structural disease in 56.3% of case, while the probability decreases to 15.2% and 5.8%, for Tg values lower than 35 and 7.1 ng/mL, respectively

The percentages of analytic cycles in which each variable was selected as predictive of persistent disease in PT-WBS was estimated. Variables selected in more than 95% of cycles were retained in the subsequent analysis. Accordingly, Tg values and N stage were the best predictive parameters in the first analytic round recurring in 100% and 97.4% of 1000 iterative cycles of analysis, respectively (Table 3). In the second step, the 200-fold cross-validation analysis was performed and the algorithm generated a conditional inference tree with five terminal nodes using Tg and N stage as predictive variables as depicted in Fig. 1. According to the decision tree model, in the first node, the N stage identifies a partition of the initial population into two subgroups according to the presence or absence of lymph node involvement. In case of lymph node involvement, patients with a value of Tg higher than 23.3 ng/mL carry a probability of 83% to have persistent/metastatic disease at PT-WBS compared to those with lower Tg values. On the other hand, patients without lymph node involvement are stratified in three risks subgroups according to their Tg values. Particularly, in the absence of lymph node involvement, Tg values exceeding 35 ng/mL predict a positive PT-WBS result in 56.3% of case, while the probability decreases to 15.2% and 5.8%, for Tg values lower than 35 and 7.1 ng/mL, respectively. Our model remained stable and reproducible in the iterative process of cross-validation just showing negligible variations in Tg threshold values selected according to the different patient groups considered by each iteration. The medians and interquartile ranges (IQR) of the Tg thresholds in different nodes are reported in the Table 4. In the training cohorts, the mean accuracy, PPV, and NPV of the generated predictive model were 88%, 68%, and 90%, respectively. Similar performance was also obtained in the validation sets, with accuracy of 88%, PPV of 60%, and NPV of 91% (Table 5). Finally, as summarized in Table 6, the accuracy, NPV, PPV, and AUC values are similar for each center with the exception of center 3 which has a lower PPV compared to other ones likely due to the use of the Roche Tg assay which produces higher results compared to Tg assays employed in other centers [23].

**Table 3:** Decision tree model: variables selected in the first analysis as predictive of post-treatment whole-body scintigraphy results were retained (enlisted in the table) and further analyzed in a cycle of iterative analysis (i.e., 1000 iterative cycles). Tg values and lymph node status (N) emerged as the best predictive parameters with a percentage of selection of 100% and 97.4%, respectively. The remaining ones, including ATA risk classes, showed a null to very low percentage of selection

Variable	% of selection
Tg	100
N	97.4
Sex	19
T	16.8
Histology	7.4
ATA risk	3.4
TSH	2.1
Age	0
RAIU	0

Selection rates  > 95% identify best predictive variables

Legend: *ATA*, American Thyroid Association; *T*, tumor; *N*, lymph node; *RAIU*, 24-h radioiodine uptake; *TSH*, thyroid stimulating hormone; *Tg*, thyroglobulin

**Table 4:** Thyroglobulin median values and inter-quartile ranges in different nodes obtained by varying the datasets during the 200-fold cross-validation analysis (training set)

Node	Median (IQR)
Node 2	35 (32.7–35)
Node 3	7.1 (6.9–7.1)
Node 7	23.3 (23.3–29.1)

Legend. *IQR*, inter-quartile range

**Table 5:** Predictive values and accuracy of thyroglobulin (median values and inter-quartile ranges) obtained during the 200-fold cross-validation analysis between training set and validation set

	Training set	Validation set
PPV	68% (67–70%)	60% (55–65%)
NPV	90% (89–90%)	91% (89–91%)
Accuracy	88% (87–88%)	88% (88–89%)

Legend: *PPV*, positive predictive value; *NPV*, negative predictive value

**Table 6:** Predictive values, accuracy, and overall performance of decision tree model and prevalence of positive post-treatment whole-body scintigraphy, respectively, in different centers and the overall population

Decision tree model	Center 1 (*n* = 208)	Center 2 (*n* = 208)	Center 3 (*n* = 83)	Center 4 (*n* = 371)	Center 5 (*n* = 447)	All (*n* = 1317)
PPV	66.7%	49.9%	18.1%	59.98%	86.6%	67%
NPV	87.6%	95.9%	95.9%	86.46%	87.34%	89.9%
Accuracy	84.6%	93.3%	85.6%	87.88%	87.27%	87.8%
AUC	0.708	0.699	0.642	0.610	0.708	0.680
Positive PT-WBS (%)	20.2%	6.7%	6%	13.2%	20.1%	15.2%

Legend: *PT-WBS*, post-treatment whole-body scintigraphy; *PPV*, positive predictive value; *NPV*, negative predictive value; *AUC*, area under the curve

## Discussion

We developed and validated an algorithm to predict whether viable tumor lesions after surgery will be visualized by PT-WBS. Notably, it should be intended as a tool to better select patients that will profit from a more intense treatment with curative intent rather than to “per se” exclude patients from ^131^I application. Currently, no definitive data exist to safely support the omission of ^131^I adjuvant treatment in low to intermediate risk DTC. Therefore, clinical decisions should include local factors, patients’ values, and preferences in addition to the conventional risk stratification. As the main result of our study, the combination of lymph node status and Tg values outperformed any other tested factor (i.e., age, sex, T, histology, ATA risk classes, TSH, and RAIU values) in predicting the presence of persistent disease after surgery. This highlights the drawback of ATA risk stratification system alone in predicting postoperative persistent disease and remarks the role of a more sophisticated postoperative assessment of DTC patients [2, 24]. Basing on our data, a decision tree is provided to guide the clinical decision-making dependent on the presence of lymph node involvement and, subsequently, on Tg levels in a different subset of patients. Notably, differences in patient selection, surgical skills, and related post-operative RAIU values (i.e., estimates of thyroid tissue remnant) and preablation TSH levels are common in clinical real life as well as the use of different Tg and TgAb assays in different centers. Overall, these factors represent a major limitation in selecting general thresholds and decision limits to inform postoperative clinical actions. Interestingly, however, neither RAIU values nor TSH levels were independently retained in our model and the impact of different Tg assays was negligible in our analysis. Accordingly, our Tg nodal thresholds were proved to be actionable even in different local populations that represent a relevant result of our study. Some limitations of our study must be mentioned. First, a potential drawback of our study is the lack of an external dataset for model validation. Rather, we performed an internal cross-validation procedure. On the other hand, this method reduces the risk of overfitting and provides a more robust estimate of model’s performance since all data are used for both training and validation. In addition, most analyzed parameters significantly varied between different centers supporting the use of an internal cross-validation instead of an external one. All in all, a more homogeneous population was obtained reflecting the real-life distribution of the parameters. Second, different Tg assays were employed in different centers and the PPV was lower in one center where a Tg assay was used which produces higher results compared to other assays [23]. However, the overall performance of the model remained good when retested in each subgroup. This is likely related to the good alignment of Tg assays employed in other participating centers. Additionally, Tg concentrations in our patients were significantly higher than those usually measured during the long term follow-up of cured DTC patients (i.e., 0.1–1 ng/mL), making the clinical impact of such analytical differences less relevant. Anyway, a careful evaluation of local Tg assay is advised, before adopting our decisional model in clinical practice [3, 4, 16, 23]. Third, our model included only patients treated after THW as most patients were treated with this preparation protocol in our centers. A significant positive correlation exists between Tg values measured under thyroxine, after rhTSH stimulation and after THW, respectively, with a basal/rhTSH-Tg and THW-Tg ratios of 1:5 and 1:10, respectively. Notwithstanding, our results cannot be directly translated to patients under thyroxine or those stimulated by rhTSH and further specific studies are warranted [16, 25, 26]. Finally, our model is explicitly intended as a tool to inform curative ^131^I administration in patients with high probability of persistent structural disease, independently by the initial risk stratification [27]. Notably, adjuvant therapy with ^131^I could still decrease the recurrence risk even in intermediate-risk patients with unstimulated Tg  ≤ 1 ng/mL or stimulated Tg  ≤ 10 ng/mL [28] making our system not actionable to rule out adjuvant ^131^I administration in low- and intermediate-risk DTC, respectively.

## Conclusions

In conclusion, we developed a simple, accurate and reproducible decision tree model able to provide reliable information on the probability of persistent/metastatic DTC after surgery. The information provided by our model is highly relevant to refine the initial risk stratification and guide ^131^I administration with adjuvant or therapeutic basing on the probability of persistent and/or metastatic disease. 

## Data Availability

The datasets generated during and/or analyzed during the current study are available from the corresponding author on reasonable request.
